# Evaluation of a population-wide, systematic screening initiative for tuberculosis on Daru island, Western Province, Papua New Guinea

**DOI:** 10.1186/s12889-024-17918-y

**Published:** 2024-04-04

**Authors:** Paison Dakulala, Margaret Kal, Alice Honjepari, Lucy Morris, Richard Rehan, Simon Peter Akena, Andrew J. Codlin, Narantuya Jadambaa, Tauhid Islam, Manami Yanagawa, Fukushi Morishita

**Affiliations:** 1 National Department of Health, Port Moresby, Papua New Guinea; 2 Western Provincial Health Authority, Daru, Papua New Guinea; 3 World Health Organization Representative Office for Papua New Guinea, Port Moresby, Papua, New Guinea; 4 World Vision International, Stop TB Programme, Daru, Papua New Guinea; 5 Friends for International TB Relief (FIT), Ho Chi Minh City, Viet Nam; 6 Department of Global Public Health, WHO Collaboration Centre on Tuberculosis and Social Medicine, Karolinska Institutet, Stockholm, Sweden; 7 Global Tuberculosis Programme, World Health Organization, Geneva, Switzerland; 8 World Health Organization Regional Office for the Western Pacific, Manila, Philippines

**Keywords:** Tuberculosis, Screening, Papua New Guinea

## Abstract

**Background:**

A population-wide, systematic screening initiative for tuberculosis (TB) was implemented on Daru island in the Western Province of Papua New Guinea, where TB is known to be highly prevalent. The initiative used a mobile van equipped with a digital X-ray device, computer-aided detection (CAD) software to identify TB-related abnormalities on chest radiographs, and GeneXpert machines for follow-on diagnostic testing. We describe the results of the TB screening initiative, evaluate its population-level impact and examine risk factors associated with TB detection.

**Methods:**

Through a retrospective review of screening data, we assessed the effectiveness of the screening by examining the enrolment coverage and the proportion of people with TB among screened subjects. A cascade analysis was performed to illustrate the flow of participants in the screening algorithm. We conducted univariate and multivariate analyses to identify factors associated with TB. Furthermore, we estimated the number of additional cases detected by the project by examining the trend of routine TB case notifications during the intervention period, compared to the historical baseline cases and trend-adjusted expected cases.

**Results:**

Of the island’s 18,854 residents, 8,085 (42.9%) were enrolled and 7,970 (98.6%) had chest X-ray interpreted by the CAD4TB software. A total of 1,116 (14.0%) participants were considered to have abnormal CXR. A total of 69 Xpert-positive cases were diagnosed, resulting in a detection rate of 853 per 100 000 population screened. 19.4% of people with TB had resistance to rifampicin. People who were in older age groups (aOR 6.6, 95%CI: 1.5–29.1 for the 45–59 age group), were severely underweight (aOR 2.5, 95%CI:1.0-6.1) or underweight (aOR 2.1, 95%CI: 1.1–3.8), lived in households < 5 people (aOR 3.4, 95%CI:1.8–6.6) and had a past history of TB (aOR 2.1, 95%CI: 1.2–3.6) were more likely to have TB. The number of bacteriologically confirmed TB notified during the intervention period was 79.3% and 90.8% higher than baseline notifications and forecasted notifications, respectively.

**Conclusion:**

The screening project demonstrated its effectiveness with the high Xpert-positive TB prevalence among the participants and by successfully yielding additional cases of bacteriologically confirmed TB including rifampicin-resistant TB. The results and lessons learnt from the project should inform future TB screening initiatives in Papua New Guinea.

**Supplementary Information:**

The online version contains supplementary material available at 10.1186/s12889-024-17918-y.

## Background

Tuberculosis (TB) continues to be one of the most impactful infectious diseases globally, with estimated 10.6 million people developing the disease and 1.4 million dying globally in 2021 [1]. The United Nations High-Level Meeting on the fight against TB in September 2018 endorsed a political declaration committing to diagnosing and treating 40 million people with TB by 2022, with an emphasis on the importance of detecting people with TB missed by existing health systems [2]. However, the COVID-19 pandemic disrupted progress against this ambitious target. TB treatment coverage rates declined sharply in 2020 and 2021, such that an estimated 40% of people with TB went undiagnosed, untreated and/or unreported globally [1].

Routine TB services are essential for those who seek care at health facilities; however, they are inadequate to find all people with TB, because services are not always accessible to poor and vulnerable populations. In addition, people with sub-clinical TB (no symptoms or symptoms not yet recognized) are also missed by routine services, as these individuals do not yet have a reason to seek out TB screening services [3]. Systematic screening, often called active case finding, targeting high-risk populations has been conducted in many countries to complement the TB services provided by the health system and to diagnosed people with TB earlier in their disease course.

The World Health Organization (WHO) recommends systematic screening for TB and has published guidelines to facilitate its implementation [4, 5]. Systematic screening is strongly recommended for people living with HIV, household contacts and other close contacts, people exposed to silica (mainly some miners), and people in prisons. In addition, systematic screening is conditionally recommended among the general population in areas with high TB prevalence and among subpopulations with structural risk factors for TB, including urban poor communities, homeless communities, communities in remote or isolated areas, indigenous populations, migrants, refugees, and other vulnerable communities with limited access to healthcare.

Papua New Guinea (PNG) is among the 30 high TB and multidrug-resistant TB (MDR-TB) burden countries worldwide. In 2021, the estimated TB incidence in PNG was 424 cases per 100,000 population, with 42,000 incident cases. An estimated proportion of MDR-TB or rifampicin-resistant TB (MDR/RR-TB) was 4.0% among new cases and 23% among previously treated cases in 2021. The Western Province, which is the largest and most sparsely populated province in PNG, had a TB case notification rate (CNR) of 674 per 100,000 in 2016 [6]. The provincial capital, Daru, an island located in the South Fly District (SFD), was considered a hotspot for both TB and MDR-TB based on previously conducted surveys and subsequent reports of TB among PNG nationals diagnosed in the Australian Torres Strait Islands, bordering SFD [7, 8]. In 2014, the CNR at the Daru General Hospital, which is the only facility providing TB services in SFD, was extremely high, with 1,031 per 100,000 population, and the number of MDR-TB cases was 84 [8], which accounted for one-fourth of the total number of people treated for MDR-TB in the country in the same year.

In response to the high burden of TB and MDR-TB in Daru, the PNG National Department of Health established the Emergency Response Taskforce in 2014 and implemented programmatic interventions until December 2017, including resource mobilisation, community engagement and strengthening TB case detection and treatment [8], which reduced the CNR to 736 per 100,000 population in 2017. However, the proportion of people with MDR-TB increased, potentially due to the introduction of GeneXpert testing as the front-line test.

The following year, the emergency response team of the National Department of Health launched the Systematic Screening Initiative (SSI) on Daru island to find and treat individuals with TB in the community. The SSI project aimed to screen the entire population of Daru island using a mobile van equipped with GeneXpert machines and a digital X-ray configured with computer-aided detection (CAD) software. In this paper, we present summary results of the TB screening activities and evaluate the effectiveness and population-level impact of the SSI project.

## Methods

### Programmatic information

PNG has a decentralized healthcare system where provincial and local governments provide TB services under policies set by the National Department of Health. In Western Province, TB control is led by the Provincial Health Officer, district health services, and Daru General Hospital, which serves as the basic management unit for TB prevention and care services. The programmatic management of drug-resistant TB was introduced in 2011 and has since strengthened over time. Daru is home to an estimated population of approximately 20,000 people. The island has a low-income economy with limited employment opportunities. It faces challenges such as poor infrastructure, lack of access to clean water and sanitation, and high rates of poverty and malnutrition.

Prior to the main implementation, the project team conducted a household survey to identify potential participants for TB screening. Community mobilization was conducted through community leaders, church leaders, businesses, and schools. Families were mapped ward by ward, and mass awareness campaigns were conducted to educate the population about TB and the importance of screening. The team also recruited and trained an operation team that included radiographers, laboratory technicians, and data collectors.

The project team used a screening van mounted on a truck equipped with digital X-ray and CAD4TB software (Delft Imaging, The Netherlands) for screening. The van was stationed near each screening site for approximately two months. Individuals were interviewed and their weight and height were recorded. They were then screened by digital chest X-ray (CXR) with CAD4TB, and if abnormalities (defined as a CAD4TB score ≥ 40) were detected, sputum samples were collected and transported to Daru General Hospital and tested using the Xpert MTB/RIF or Ultra assay regardless of the presence of symptoms. The threshold of a CAD4TB score was a value recommended by the manufacturer during the pilot stage; then it was readjusted to 40 to make it more sensitive during the main implementation phase. If CXR findings were considered normal (a CAD4TB score < 40), yet the person is symptomatic, sputum samples were collected for the Xpert. If Xpert results were MTB negative, no further action was taken. Clinical diagnoses of pulmonary TB and extra-pulmonary TB were not made as part of this project. Those with a positive Xpert result were considered bacteriologically-confirmed pulmonary TB, and appropriate TB treatment was provided and managed by the Daru General Hospital, following the national guidelines. Sputum samples from those with RR-TB were sent to the Central Public Health Laboratory for culture, drug susceptibility testing (DST), and line probe assay.

According to the protocol, the screening initiative included people aged 10 years and above, who reside on the island and were willing and able to come to the mobile clinic or hospital, and who provided informed consent. Pregnant women, children below 10 years old, severely ill individuals who could not visit the mobile clinic or hospital, and individuals who had already been diagnosed with TB and were on treatment were excluded from the screening. In the actual field setting, however, children aged between five and nine who were willing to participate were also screened upon the request of their guardians. Children aged 0–4 were completely excluded since it is difficult to collect sputum specimen The exclusion of pregnant women was based on concerns about potential fetal harm due to radiation exposure. The informed consent was obtained from all subjects and/or their legal guardians. The field staff explained the form verbally to all participants and read the exact words or those who had difficulties in reading the text. If they couldn’t write their signatures, they were allowed to ask others to delegate and give their thumbprint. If the participant was a minor, informed consent was obtained from their guardians. Participants were informed that their participation was entirely voluntary and that provided information would be kept confidential. The ethical approval was obtained from the Ethics Board of the Medical Research Advisory Committee at the National Department of Health in Papua New Guinea.

The screening was conducted in two phases. The main implementation period was from February to October 2018, and the mop-up screening was organized from February to March 2019 to invite and screen those who were missed from the main implementation period. Around 30–80 people were screened per day during the main implementation phase. The screening took place on weekdays throughout the main implementation periods, with weekends being included for mop-up screenings.

### Quantitative data and statistical analysis

The data collected during the project were managed using paper forms which were later digitized. The data collection forms included variables for sex, age, ward, household size, housing type, education, occupation, income, behavioral and social risk factors, symptoms and CAD4TB score. These data were entered into Microsoft Access to develop a participant-level dataset for analysis. Additionally, the aggregate data, such as the number of people screened, tested and diagnosed with TB, were recorded and stored in Microsoft Excel.

The effectiveness of the screening project was assessed by ward using the enrolment coverage and the proportion of people diagnosed with TB (or a detection rate per 100,000 population) among screened subjects, which was used as a proxy for TB prevalence. The number needed to screen (NNS) to detect a person with TB was also calculated as part of the effectiveness analysis. Cascade analysis was performed using participant-level data to illustrate the completion of the screening algorithm. We then performed a univariate analysis to identify risk factors for TB. Using the variables deemed significant in the univariate analysis, a multivariate logistic regression analysis was performed to confirm the observed association. Considering a higher likelihood of false-positive Xpert results among individuals with a past history of TB compared to those without a past history of TB, we conducted a supplementary analysis to validate the results of the risk factor analysis by removing those with a past history of TB (*n* = 1068) and repeating the analysis. Odds ratios (OR) for univariate analysis and adjusted odds ratios (aOR) for multivariate analysis were calculated to examine the strength of the association, along with a 95% Confidence Interval (CI). Statistical significance was set at *p* < 0.05. Data processing and statistical analyses were performed using Stata 13 (StataCorp, College Station, TX, USA).

Finally, we quantified the additional notifications above trend-expected TB case notification from Daru island, disaggregated by drug-resistance status [9]. Drug-sensitive TB notification policy changed in 2017, and notification data from 2017-Q4 (just before the SSI project started) were submitted in a different format from the notification data submitted in the past. Therefore, the old and new notification formats were mapped to construct a continuous time series. In routine case notification data, bacteriologically confirmed TB refers to a case from whom a biological specimen is positive by Xpert, culture or smear microscopy.

## Results

### Effectiveness analysis

Of the estimated population of Daru island, 42.9% (*n* = 8,085/18,854) were enrolled in the project and screened for TB. Of those agreeing to participate, 0.85% (*n* = 69/8085) were diagnosed with TB, which translates to a NNS of 117 (Table 1). The enrolment coverage and yields were variable across the four wards. Karakara ward achieved the highest population coverage rate (56.2%), followed by Darowaro (44.3%), Iaru (34.8%) and Tamati (31.2%). The highest TB detection rate was in Iaru ward (1,401 per 100 000 population), followed by Darowaro (1,064 per 100 000 population), Tamati (892 per 100 000 population) and Karakara (461 per 100 000 population).

**Table 1:** Population coverage of SSI project and yield of TB screening by ward

	Darowaro	Iaru	Karakara	Tamati	All Wards
**Estimated population**	3 604	5 132	6 168	3 950	18 854
**Enrolled**	1 598 (44.3%)	1 785 (34.8%)	3 469 (56.2%)	1 233 (31.2%)	8 085 (42.9%)
**Screened by CXR**	1 539 (96.3%)	1 770 (99.2%)	3 448 (99.4%)	1 213 (98.4%)	7 970 (98.6%)
**CAD4TB score ≥ 40**	247 (16.0%)	322 (18.2%)	295 (8.6%)	252 (20.8%)	1 116 (14.0%)
**Tested with Xpert/Ultra**	215 (87%)	232 (72%)	234 (79.3%)	209 (82.9%)	890 (79.7%)
**Detected with TB**	17 (7.9%)	25 (10.8%)	15 (6.4%)	10 (4.8%)	67 (7.5%)
**Off algorithm Xpert/Ultra testing**	42	7	6	9	64
**Detected with TB off algorithm**	0 (0%)	0 (0%)	1 (16.7%)	1 (11.1%)	2 (3.1%)
**Total detected with TB**	17	25	16	11	69
**TB case detection rate per 100 000 population**	1 064	1 401	461	892	853
**NNS**	94	71	217	112	117

### Cascade analysis

**Fig. 1:**
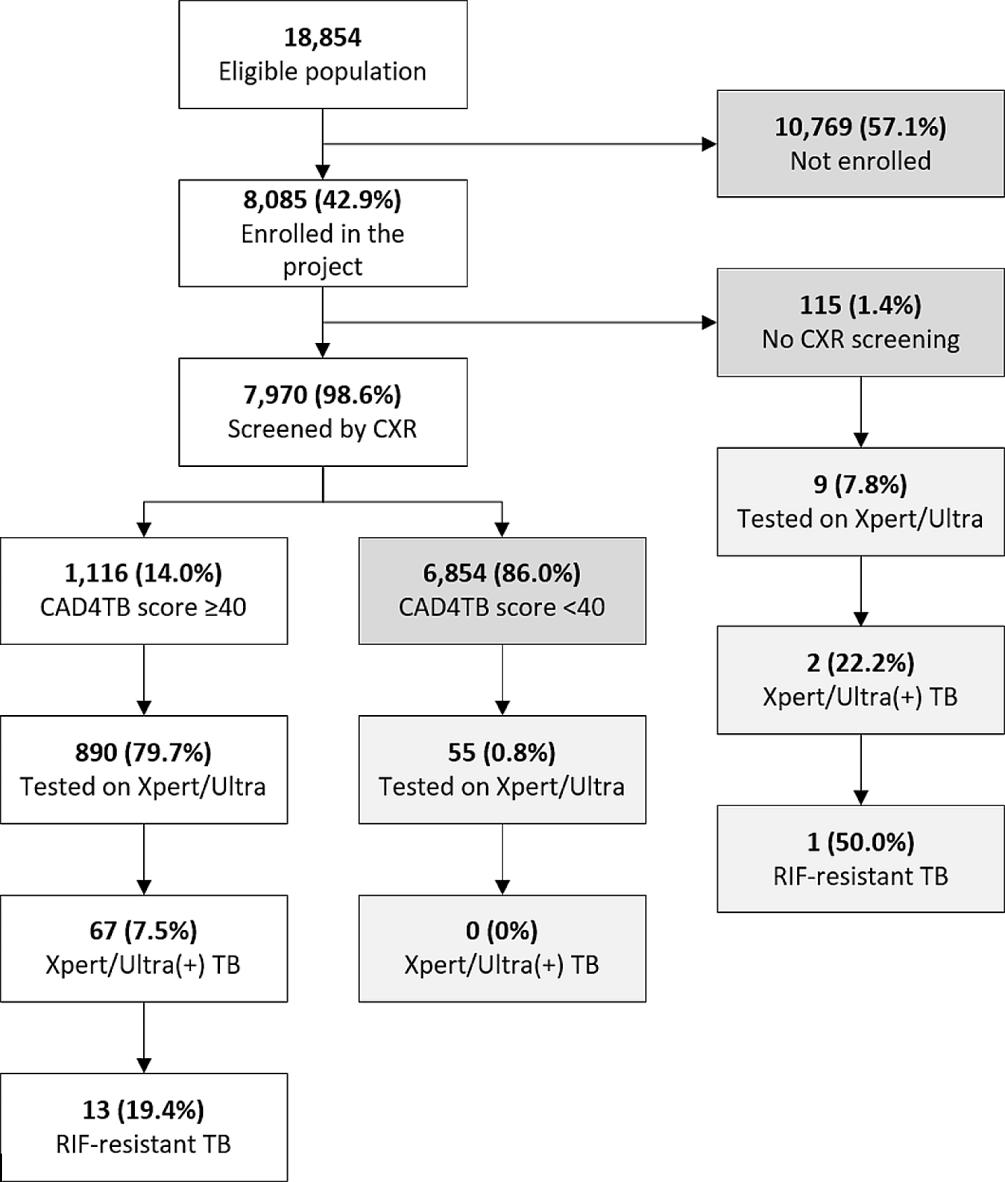
Project yield along the cascade of care for TB screening

Of 8,085 participants recorded in the participant-level dataset, 98.6% (*n* = 7,970) were screened by CXR and 14.0% (*n* = 1,116) had an abnormal CXR result (Fig. 1). Of those with an abnormal CXR, 79.7% (*n* = 890) were tested using Xpert, resulting in the detection of 67 (7.5%) people with bacteriologically confirmed TB. Of these, 19.4% (*n* = 13) were resistant to rifampicin. The proportion of people diagnosed with TB who reported any symptoms suggestive of TB was 6.7% (Table 1). Of 115 participants without CXR result, 7.8% (*n* = 9) were tested by Xpert, resulting in the detection of two additional people with TB (22.2% positivity rate) including one person who had resistance to rifampicin. Of 6,954 participants with a CAD4TB score < 40, 0.8% (*n* = 55) were tested by Xpert, resulting in no case detection.

### Univariate and multivariate analysis of risk factors

Statistically significant factors associated with TB identified by the multi-variate analysis were age group, body mass index (BMI), Ward, household size, and a past history of TB (Table 2). Males were marginally more likely than females to be diagnosed with TB (aOR 1.6, 95% CI: 1.0-2.8). There was a step-wise increase in odds with increasing age groups, with the highest odds found in the 45–59 age group (aOR 6.6, 95% CI: 1.5–29.1). Individuals with severe underweight were 2.5 times more likely to have TB (aOR 2.5, 95% CI: 1.0-6.1) than those with a normal weight, while people with overweight were significantly less likely to have TB (aOR 0.1, 95% CI: 0.01–0.7). Residents of specific geography (Iaru ward) had a higher chance of having TB (aOR 2.1, 95% CI: 1.0-4.2). Smaller household size < 5 people was associated with over three times increased odds of TB (aOR 3.4, 95% CI: 1.8–6.6). No significant differences were observed in odds of TB across the income groups after adjustment for covariates. People who had a past history of TB were more likely to have TB (aOR 2.1, 95% CI: 1.2–3.6), but those with symptoms only had a marginally higher risk after adjustment (aOR 1.8, 95% CI: 0.9–3.5). These results remain consistent in the supplementary analysis that excluded those without a past history of TB (Supplementary Material).

**Table 2:** Univariate and multivariate analyses of clinical, demographic, behavioural and socioeconomic risk factors for TB

Characteristics		Total	Confirmed TB Case (%)	Univariate	Multivariate
				Crude OR (95% CI)	Adjusted OR (95% CI)
Sex	Male	3 852 (50.3%)	42 (1.1%)	1.72 (1.04–2.85)*	1.64 (0.95–2.81)
	Female	3 777 (49.3%)	24 (0.6%)	1.00 (Ref)	1.00 (Ref)
	Unknown	25 (0.3%)	0 (0.0%)		
Age Group	5–14 yrs	994 (13.0%)	2 (0.2%)	1.00 (Ref)	1.00 (Ref)
	15–29 yrs	3 026 (39.5%)	21 (0.7%)	3.47 (0.81–14.81)	3.17 (0.71–14.16)
	30–44 yrs	1 808 (23.6%)	18 (1.0%)	4.99 (1.15–21.54)*	3.71 (0.81–16.89)
	45–59 yrs	1 348 (17.6%)	22 (1.6%)	8.23 (1.93–35.08)*	6.57 (1.48–29.13)*
	≥ 60 yrs	478 (6.3%)	3 (0.6%)	3.13 (0.52–18.81)	2.21 (0.36–13.59)
BMI	Severely Underweight	268 (5.1%)	6 (2.2%)	2.78 (1.17–6.60)*	2.46 (1.00-6.14)*
	Underweight	883 (25.6%)	17 (1.9%)	2.38 (1.35–4.20)*	2.07 (1.14–3.76)*
	Normal weight	5 138 (52.9%)	42 (0.8%)	1.00 (Ref)	1.00 (Ref)
	Overweight	1 365 (16.4%)	1 (0.1%)	0.09 (0.01–0.65)*	0.09 (0.01–0.65)*
Ward	Karakara	3 368 (44.0%)	15 (0.4%)	1.00 (Ref)	1.00 (Ref)
	Tamati	1 134 (14.8%)	9 (0.8%)	1.79 (0.78–4.10)	1.08 (0.45–2.60)
	Darowaro	1 486 (19.4%)	17 (1.1%)	2.59 (1.29–5.19)*	1.67 (0.80–3.48)
	Iaru	1 666 (21.8%)	25 (1.5%)	3.41 (1.79–6.48)*	2.09 (1.04–4.20)*
Household Size	< 5 people	614 (8.0%)	16 (2.6%)	4.00 (2.14–7.47)*	3.41 (1.78–6.55)*
	5–9 people	2 974 (38.9%)	23 (0.8%)	1.17 (0.67–2.04)	1.09 (0.62–1.92)
	≥ 10 people	4 066 (53.1%)	27 (0.7%)	1.00 (Ref)	1.00 (Ref)
Housing Type	Individual house	5 506 (71.9%)	46 (0.8%)	1.00 (Ref)	
	Settlement, Prison & Unknown	2 148 (28.1%)	20 (0.9%)	1.11 (0.67–1.89)	
Education	None	265 (3.5%)	4 (1.5%)	2.39 (0.81–7.05)	
	Elementary	4 246 (55.5%)	42 (1.0%)	1.56 (0.91–2.66)	
	Secondary and higher, or unknown	3 143 (41.1%)	20 (0.6%)	1.00 (Ref)	
Job	None	3 564 (46.6%)	31 (0.9%)	1.00 (Ref)	
	Formal employment	3 324 (43.4%)	27 (0.8%)	0.93 (0.56–1.57)	
	Informal employment & Unknown	766 (10.0%)	8 (1.0%)	1.20 (0.55–2.63)	
Income (kina/month)	< 200	3 810 (49.8%)	42 (1.1%)	2.58 (1.32–5.01)*	1.69 (0.83–3.41)
	200–499	1 011 (13.2%)	9 (0.9%)	2.08 (0.86–5.02)	1.46 (0.59–3.69)
	≥ 500	2 553 (33.4%)	8 (0.3%)	1.00 (Ref)	1.00 (Ref)
	Unknown	280 (3.7%)	4 (1.4%)	3.35 (1.06–10.59)*	2.23 (0.67–7.41)
Presence of behavioral and social risk factors and symptoms	Smoking	2 700 (35.3%)	36 (1.3%)	2.21 (1.36–3.60)*	1.37 (0.80–2.34)
	Alcohol	2 367 (30.9%)	26 (1.1%)	1.45 (0.88–2.39)	
	Buai	4 204 (54.9%)	38 (0.9%)	1.11 (0.68–1.81)	
	Cough	265 (3.5%)	8 (3.0%)	3.93 (1.86–8.31)*	1.77 (0.9–3.47)†
	Fever	166 (2.2%)	9 (5.4%)	7.46 (3.63–15.34)*	
	Night Sweats	164 (2.1%)	7 (4.3%)	5.70 (2.56–12.69)*	
	Loss of Appetite	145 (1.9%)	4 (2.8%)	3.40 (1.22–9.47)*	
	Past history of TB	1 068 (14.0%)	23 (2.2%)	3.34 (2.00-5.56)*	2.10 (1.22–3.63)*
	Household contact	753 (9.8%)	11 (1.5%)	1.84 (0.96–3.53)	
	Social contact	715 (9.3%)	8 (1.1%)	1.33 (0.63–2.80)	
CAD4TB Score	< 50	7 066 (92.3%)	12 (0.2%)	1.00 (Ref)	
	50–59	219 (2.9%)	12 (5.5%)	34.08 (15.13–76.76)*	
	60–69	175 (2.3%)	17 (9.7%)	63.25 (29.71-134.65)*	
	70–79	98 (1.3%)	12 (12.2%)	82.02 (35.84-187.72)*	
	80–89	45 (0.6%)	9 (20.0%)	146.96 (58.32-370.32)*	
	90–100	51 (0.7%)	4 (7.8%)	50.03 (15.57-160.77)*	

CI: Confidence interval, OR: Odds Ratio

* Significant difference (*p* < 0.05)

† Multivariate adjusted OR was calculated using the single yes/no binary variable for any symptom (cough, fever, loss of appetite, and/or night sweats)

### Additionality analysis

**Fig. 2:**
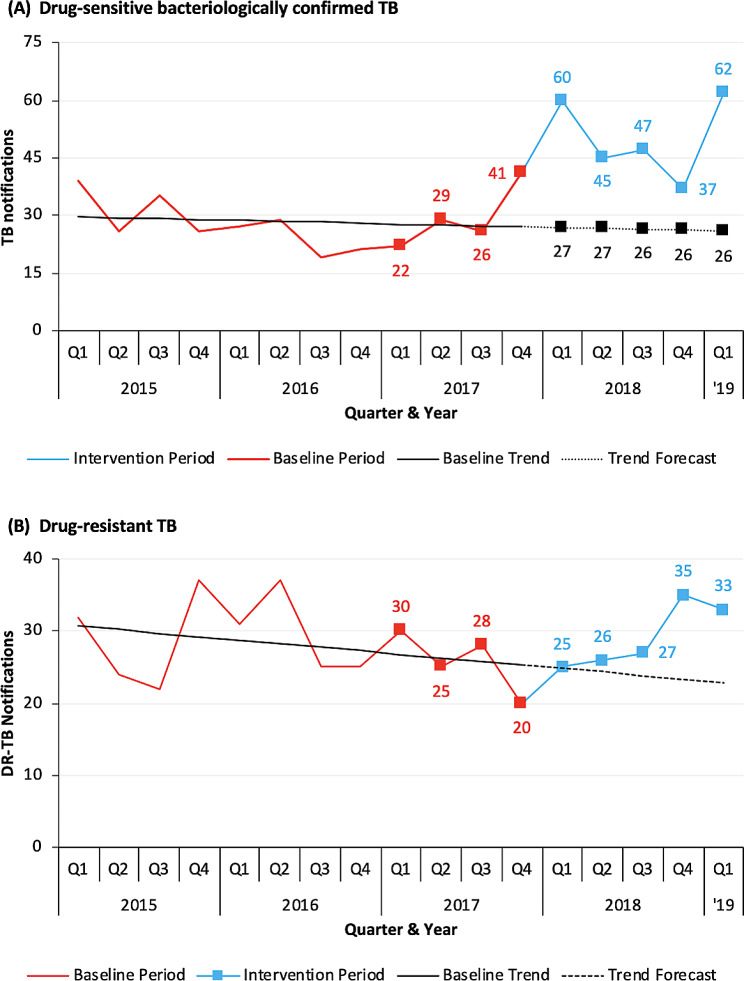
TB notifications at the Daru Hospital by quarter

The total number of all forms drug-sensitive TB notifications during the project implementation period was 576, which was − 13.3% and − 3.2% lower than baseline period notifications (*n* = 664) and forecasted notifications (*n* = 595), respectively (Table 3). On the other hand, the total number of bacteriologically confirmed TB notifications (*n* = 251) was + 79.3% and + 90.8% higher than baseline period notifications (*n* = 140) and forecasted notifications (*n* = 132), respectively. As compared to baseline period notifications and forecasted notifications, the number of clinically diagnosed TB notifications was lower (-77.9% and − 78.5%, respectively), and that of extra-pulmonary TB notifications was considerably higher (+ 101.7% and + 200%, respectively). A total of 146 drug-resistant TB (DR-TB) patients were notified during the project implementation period. This was + 9.8% and + 22.5% higher than baseline period notifications (*n* = 133) and forecasted notifications (*n* = 119), respectively. The quarterly trends of case notifications for bacteriologically confirmed drug-susceptible TB (DS-TB) and DR-TB and the comparisons with baseline notifications and forecasted notifications are provided in Fig. 2.

**Table 3:** Changes in TB notification data at the Daru Hospital by type of TB

		DS-TB						All DR-TB
		All Bac(+)	All CD	All PTB	All EP	Other	All Forms	
Implementation period* notifications		251	85	336	240	0	576	146
Pre/Post Analysis	Baseline period** notifications	140	384	524	119	21	664	133
	Additional notifications	111	-299	-188	121	-21	-88	13
	Percent change from baseline	+ 79.3%	-77.9%	-35.9%	+ 101.7%	-100.0%	-13.3%	+ 9.8%
Trend Regression Analysis	Forecasted implementation notifications	132	395	525	80	-15	595	119
	Additional notifications	119	-310	-189	160	15	-19	27
	Percent change from forecast	+ 90.8%	-78.5%	-36.0%	+ 200.0%	-100.0%	-3.2%	+ 22.5%

* 2018-Q1 to 2019-Q1

** (2017-Q1 × 2) + 2017-Q2 to 2017-Q4

Note: All Bac(+) includes new, relapse, treatment after failure, treatment after default, previously treated, other and unknown cases. All CD includes the clinically diagnosed categories of smear-negative and ‘not done / not available’ for all treatment histories. All PTB is the sum of pulmonary Bac(+) and clinically diagnosed notifications. All EP includes extrapulmonary TB notifications for all treatment categories. All Forms is the sum of all notification categories, including other

## Discussion

The results from our analyses revealed various achievements and challenges in the SSI project. The overall TB detection rate among the project participants was high, with 853 per 100,000. This was higher than the estimated national incidence for PNG in 2019 (432 per 100,000 population) [10] and well above the minimum threshold for recommending community-wide systematic screening (500 per 100,000 population) [5], which demonstrated the effectiveness of the project. NNS of 117 were comparable with other similar community-wide systematic screening projects in high-incidence settings [11].

While the Xpert-positive TB prevalence was high in the project, it was substantially lower than the CNR on the island during the project implementation period, and well below peaks of historical CNR. Several factors could explain this difference. Firstly, while the project focused on screening residents on Daru island, the Daru General Hospital caters TB services to those travelling from the mainland too. This could have affected the comparability. Secondly, this project focused on detecting bacteriologically confirmed pulmonary TB in individuals aged 10 or over, while the reported CNR included clinically diagnosed TB, extrapulmonary TB and TB in children under 10 years old. This difference could also account for our finding that the proportion of additional cases was high for bacteriologically confirmed TB. It is notable that the number of additional bacteriologically confirmed TB notifications was greater than the direct yield of the project, while the case notification of clinically diagnosed TB during the intervention period was lower than baseline and forecasted cases. This may be due to the change in notification policy implemented in 2017-Q4, where individuals with smear-negative, Xpert-positive results are reported as bacteriologically confirmed TB while it was reported as smear-negative TB in the past.

Our additionality analysis showed that the number of extrapulmonary TB, which was not the primary focus of the project, increased considerably during the intervention period. In general, community-wide screening raise awareness about TB and increases health-seeking behaviour in communities [12, 13]. Children under 10 years old who are not eligible for participation might have visited the hospital voluntarily, which could have increased routine case notification of extrapulmonary TB since paediatric TB is more likely to be extrapulmonary than adult [14].

One of the achievements of the project was to ensure high coverage of CXR (98.6%), following the screening algorithms in the protocol. Ensuring CXR for all can be operationally challenging in a programmatic setting, however, the combined use of digital X-ray and CAD4TB in this project replaced the human processing and interpretation of X-ray and contributed to the high CXR coverage. In this project, the proportion of people diagnosed with TB who had symptoms was very small. This suggested that most of the detected TB cases (> 80%) were asymptomatic and were captured by the contribution of CXR.

The results demonstrated that the project successfully yielded additional people with rifampicin-resistant TB compared to the baseline and forecasted notifications (9.8% and 22.5%, respectively). Around one-fifth (19.4%) of the detected TB patients had resistance to rifampicin. This was an extremely high proportion, compared to the global (4.6%) and national averages (5%) from the surveillance reports for bacteriologically confirmed TB [10]. This confirmed the fact that Daru is a hotspot of MDR/RR-TB and highlighted the importance of using Xpert testing as an initial diagnostic test, not only in the screening project, but also in the routine setting to facilitate early detection and treatment of DR-TB. Given that Daru is a place, together with Port Moresby, where extensively drug-resistant (XDR) TB (defined as MDR-TB plus resistance to any fluoroquinolone and a second-line injectable) and pre-XDR-TB (resistance to a fluoroquinolone or a second-line injectable) had been reported, ensuring rapid DST in addition to RR-TB detection is critical [15].

Our analysis found that people who were male, in older age groups, had lower BMI and had a past history of TB were more likely to have TB. While we did not find comprehensive literature on risk factors for TB in PNG, our findings were generally consistent with studies/surveys in other countries [16–18]. The relationship between BMI and TB is well-established, with numerous studies demonstrating a bidirectional association between TB and malnutrition [19–21]. In PNG, undernourishment is the major driver for TB, with an estimated 15,000 (95% CI: 12,000–18,000) TB cases attributable to undernourishment [10]. Our finding underscored the need for integrating nutritional assessments into TB services and vice versa. In this study, smaller household size was associated with a three-fold increase in the odds of TB. This is contrary to the literature, where crowding is a consistent risk factor for TB [22] and it is a known problem in Daru [23]. This could be explained if people living in larger households may spend less time in their homes, avoiding crowded conditions. This should be investigated more thoroughly.

The SSI project aimed to screen all eligible populations on Daru island. However, only 42.9% of them were enrolled in the project. Furthermore, 20% of those with abnormal CXR did not proceed to Xpert testing. Systematic TB screening using CXR as an initial screening tool involves asymptomatic individuals who think they are healthy. Coupled with a high level of social stigma around TB in the country [24], this could have resulted in the suboptimal enrolment rate and the test retention rate. The acceptability of the population-wide screening should be studied using a qualitative method to inform community mobilization strategies in future disease screening projects in PNG.

This study has several limitations. First, the patient-level database had suboptimal data quality, with duplicate entries and missing information. The laboratory data were not initially incorporated into the database, and matching was done manually using name, age, and sex variables as the constructed unique identifier. This could have affected the accuracy of the risk factor analysis. Second, the data from the off-algorithm testing could have affected the computation of process indicators and complicated the interpretation of the results from the cascade analysis. However, such flexible arrangements yielded two TB cases. Third, some of the Xpert-positive cases with a past history of TB (*n* = 23) might have false-positive Xpert results, given that Xpert detects dead TB bacilli, which could have influenced the overall screening results. Although our supplementary analysis confirmed that major risk factors remain the same among participants without a history of TB, we were unable to assess the magnitude of possible false-positive results since culture test was not performed in this project. Fourth, results from the additionality analysis might have been over or underestimated due to the issues in comparability and the change in the notification policy as mentioned above. Finally, the results from the project cannot represent the entire population in Daru since the project did not achieve full population coverage with its screening activities. It is reasonable to assume that there was a selection bias in the participants being sicker than the general population. Nevertheless, the local interpretation of the varying prevalence and patient profile by ward is encouraged to better understand the disease pattern.

Despite these limitations, this evaluation study was the first to document the results from a large-scale population-wide TB screening in PNG. Using a mobile van, the SSI project brought state-of-the-art screening tools and diagnostics to poor rural communities. Small island settings in the Pacific, where the population is geographically isolated and defined, provide a unique opportunity for population-wide screening. Such interventions are increasingly implemented as part of the effort to eliminate TB, in combination with TB infection testing and preventive treatment [25, 26]. Given the high burden of TB and the paucity of evidence on systematic TB screening in PNG, all future TB screening projects should incorporate a well-designed evaluation to add to the evidence base and inform screening strategies [27]. Operational challenges and data management issues revealed by this project should be carefully examined and addressed in future initiatives to ensure the successful implementation of TB screening programs in PNG.

### Electronic supplementary material

Below is the link to the electronic supplementary material.

Supplementary Material 1

## Data Availability

The datasets used and/or analysed during the current study are available from the corresponding author on reasonable request.

## Abbreviations

aOR: Adjusted odds ratios
BMI: Body mass index
CAD: Computer-aided detection
CD: Clinically diagnosed
CI: Confidence interval
CNR: Case notification rate
CXR: Chest X-ray
DST: Drug susceptibility testing
DS-TB: Drug-susceptible tuberculosis
EP: Extrapulmonary tuberculosis
MDR-TB: Multidrug-resistant tuberculosis
NNS: Number needed to screen
OR: Odds ratios
PNG: Papua New Guinea
PTB: Pulmonary tuberculosis
RR-TB: Rifampicin-resistant tuberculosis
SFD: South Fly District
SSI: Systematic Screening Initiative
TB: Tuberculosis
WHO: World Health Organization
XDR-TB: Extensively drug-resistant tuberculosis
