# Supplementary material for: Evaluation of a population-wide, systematic screening initiative for tuberculosis on Daru island, Western Province, Papua New Guinea
Source: BMC Public Health. 2024 Apr 4;24:959. doi: 10.1186/s12889-024-17918-y (PMC10993525; doi:10.1186/s12889-024-17918-y)
Supplement: Supplementary file 1 — Supplementary Material 1 [file 12889_2024_17918_MOESM1_ESM.pdf]

Supplementary Material

Supplementary Table. Univariate and multivariate analyses of clinical, demographic, behavioural and socioeconomic risk factors for TB excluding participants with a past history of TB

Characteristics		Total	Confirmed TB Case (%)	Univariate	Multivariate
				Crude OR (95% CI)	Adjusted OR (95% CI)
Sex	Male	3 339 (50.8%)	28 (0.8%)	1.81 (0.96-3.39)	
	Female	3 209 (48.8%)	15 (0.5%)	1.00 (Ref)	
	Unknown	23 (0.4%)	0 (0%)		
Age Group	5-14 yrs	920 (14%)	2 (0.2%)	1.00 (Ref)	1.00 (Ref)
	15-29 yrs	2 716 (41.2%)	14 (0.5%)	2.38 (0.54-10.48)	2.61 (0.56-12.16)
	30-44 yrs	1 486 (22.6%)	12 (0.8%)	3.74 (0.83-16.73)	3.27 (0.68-15.62)
	45-59 yrs	1 088 (16.5%)	14 (1.3%)	5.98 (1.36-26.39)*	6.15 (1.33-28.37)*
	≥60	376 (5.7%)	1 (0.3%)	1.22 (0.11-13.54)	0.91 (0.08-10.33)
BMI	Severely Underweight	197 (3%)	5 (2.5%)	4.16 (1.59-10.89)*	4.52 (1.66-12.32)*
	Underweight	683 (10.4%)	10 (1.5%)	2.37 (1.15-4.91)*	2.13 (1.02-4.48)*
	Normal weight	4 500 (68.3%)	28 (0.6%)	1.00 (Ref)	1.00 (Ref)
	Overweight	1 206 (18.3%)	0 (0%)		
Ward	Karakara	3 057 (46.4%)	10 (0.3%)	1.00 (Ref)	1.00 (Ref)
	Tamati	899 (13.7%)	5 (0.6%)	1.70 (0.58-5.00)	1.35 (0.45-4.07)
	Darowaro	1 252 (19%)	10 (0.8%)	2.45 (1.02-5.91)*	1.74 (0.70-4.32)
	Iaru	1 378 (20.9%)	18 (1.3%)	4.03 (1.87-8.76)*	3.10 (1.39-6.92)*
Household Size	<5 people	507 (7.7%)	10 (2%)	4.19 (1.91-9.20)*	4.07 (1.81-9.21)*
	5-9 people	2 522 (38.3%)	16 (0.6%)	1.33 (0.67-2.64)	1.24 (0.61-2.47)
	≥10 people	3 557 (54%)	17 (0.5%)	1.00 (Ref)	1.00 (Ref)
Housing Type	Individual house	4 782 (72.6%)	32 (0.7%)	1.00 (Ref)	
	Settlement, Prison & Unknown	1 804 (27.4%)	11 (0.6%)	0.91 (0.46-1.81)	
Education	None	227 (3.4%)	3 (1.3%)	2.28 (0.66-7.87)	
	Elementary	3 625 (55%)	24 (0.7%)	1.13 (0.60-2.14)	
	Secondary and higher, or unknown	2 734 (41.5%)	16 (0.6%)	1.00 (Ref)	
Job	None	3 129 (47.5%)	20 (0.6%)	1.00 (Ref)	
	Formal employment	2 802 (42.5%)	19 (0.7%)	1.06 (0.56-1.99)	
	Informal employment & Unknown	655 (9.9%)	4 (0.6%)	0.96 (0.33-2.80)	
Income (kina/month)	<200	3 235 (49.1%)	27 (0.8%)	2.35 (1.06-5.18)*	
	200-499	870 (13.2%)	6 (0.7%)	1.94 (0.67-5.60)	
	≥500	2 240 (34%)	8 (0.4%)	1.00 (Ref)	
	Unknown	241 (3.7%)	2 (0.8%)	2.33 (0.49-11.06)	
Presence of behavioral and social risk factors and symptoms	Smoking	2 319 (35.2%)	23 (1.0%)	2.12 (1.16-3.87)*	1.42 (0.75-2.69)
	Alcohol	2 022 (30.7%)	16 (0.8%)	1.32 (0.71-2.46)	
	Buai	3 594 (54.6%)	25 (0.7%)	1.13 (0.61-2.08)	
	Cough	163 (2.5%)	5 (3.1%)	5.14 (2.00-13.23)*	2.14 (0.88-5.25)†
	Fever	99 (1.5%)	5 (5.1%)	8.55 (3.30-22.18)*	
	Night Sweats	106 (1.6%)	3 (2.8%)	4.55 (1.38-14.92)*	

	Loss of Appetite	96 (1.5%)	2 (2.1%)	3.27 (0.78-13.71)	
	Household contact	597 (9.1%)	5 (0.8%)	1.31 (0.51-3.33)	
	Social contact	579 (8.8%)	4 (0.7%)	1.05 (0.37-2.94)	
CAD4TB Score	<50	6 273 (95.2%)	7 (0.1%)	1.00 (Ref)	
	50-59	144 (2.2%)	8 (5.6%)	52.66 (18.83-147.27)*	
	60-69	94 (1.4%)	13 (13.8%)	143.66 (55.86-369.48)*	
	70-79	38 (0.6%)	6 (15.8%)	167.84 (53.44-27.12)*	
	80-89	14 (0.2%)	6 (42.9%)	671.36 (184.40-2,444.30)*	
	90-100	23 (0.3%)	3 (13.0%)	134.27 (32.39-556.58)*	

CI: Confidence interval, OR: Odds Ratio

* Significant difference ($p < 0.05$)

† Multivariate adjusted OR was calculated using the single yes/no binary variable for any symptom (cough, fever, loss of appetite, and/or night sweats)
